# Cooperation and competition between the default mode network and frontal parietal network in the elderly

**DOI:** 10.3389/fpsyg.2023.1140399

**Published:** 2023-05-19

**Authors:** Hideya Koshino, Mariko Osaka, Tetsuya Shimokawa, Mizuki Kaneda, Seira Taniguchi, Takehiro Minamoto, Ken Yaoi, Miyuki Azuma, Katsuki Higo, Naoyuki Osaka

**Affiliations:** ^1^Department of Psychology, California State University, San Bernardino, CA, United States; ^2^Graduate School of Human Sciences, Osaka University, Suita, Osaka, Japan; ^3^Center for Information and Neural Networks (CiNet), National Institute of Information and Communications Technology (NICT), Suita, Osaka, Japan; ^4^Department of Psychology, Graduate School of Letters, Kyoto University, Kyoto, Japan

**Keywords:** DMN, FPN, aging, functional connectivity, working memory

## Abstract

Recent research has shown that the Default Mode Network (DMN) typically exhibits increased activation during processing of social and personal information but shows deactivation during working memory (WM) tasks. Previously, we reported the Frontal Parietal Network (FPN) and DMN showed coactivation during task preparation whereas the DMN exhibited deactivation during task execution in working memory tasks. Aging research has shown that older adults exhibited decreased functional connectivity in the DMN relative to younger adults. Here, we investigated whether age-related cognitive decline is related to a reduced relationship between the FPN and DMN using a working memory task during the execution period. First, we replicated our previous finding that the FPN and DMN showed coactivation during the preparation period, whereas the DMN showed deactivation during the execution period. The older adults showed reduced DMN activity during task preparation and reduced deactivation during task execution; however, they exhibited a higher magnitude of activation in the FPN than the young individuals during task execution. Functional connectivity analyses showed that the elderly group, compared to the young group, showed weaker correlations within the FPN and the DMN, weaker positive correlations between the FPN and DMN during task preparation, and weaker negative correlations between the FPN and DMN during execution. The results suggest that cognitive decline in the older adults might be related to reduced connectivity within the DMN as well as between the FPN and DMN.

## Introduction

In the present study, we investigated how the relationship between the Default Mode Network (DMN) and Frontoparietal network (FPN) affects age-related cognitive decline in a working memory task. The DMN typically consists of the Medial Prefrontal Cortex (MPFC), the Posterior Cingulate Cortex (PCC)/Precuneus, the Inferior Parietal Lobe (IPL), the Lateral Temporal Cortex (LTC), and the Hippocampal Formation (HF) (e.g., Gusnard and Raichle, 2001; Buckner et al., 2008; Andrews-Hanna et al., 2010; Raichle, 2015). Among these regions, the MPFC and PCC are typically considered as the core regions (hubs) of the DMN. Recent research has shown that the DMN is associated with social and personal information processing, including mind wandering, which is the experience of having one’s thought drift away from a current task, episodic memory and prospection, processing of information related to self, social cognition, and theory of mind (e.g., Gusnard and Raichle, 2001; Mason et al., 2007; Schacter et al., 2007; Spreng et al., 2008; Christoff et al., 2009; Sestieri et al., 2011; Andrews-Hanna, 2012).

However, the DMN also shows deactivation during various cognitive tasks (e.g., Shulman et al., 1997; Gusnard and Raichle, 2001; Raichle, 2015), which is viewed as a form of task induced deactivation (TID). When resource demands increase during tasks, allocation of processing resources increased in task relevant brain regions, whereas resources in task irrelevant regions would decrease, resulting in deactivation in the task irrelevant regions (e.g., Smith et al., 2004; Fox et al., 2005; Uddin et al., 2009). It has been shown also that the DMN shows anti-correlation with task positive networks during various cognitive tasks. For example, Fox et al. (2005) showed that the DMN activity decreased as the dorsal attention network (DAN) activity increased, whereas the DAN activity decreased as the DMN activity increased. Other studies showed that when people are performing cognitive tasks, the Frontal Parietal Network (FPN), including the dorsolateral prefrontal cortex (DLPFC) and the inferior parietal lobe (IPL), require more processing resources; and therefore, activity of the DMN regions decreases. (e.g., Greicius et al., 2003; Fox et al., 2005; Hampson et al., 2006; Tomasi et al., 2006; Weissman et al., 2006; Kelly et al., 2008; Keller et al., 2015; Piccoli et al., 2015).

However, subsequent research has shown that the DMN and FPN can work together, as they show co-activation during some cognitive tasks, such as autobiographical planning (e.g., Spreng and Grady, 2010), evaluation of creative activity (e.g., Ellamil et al., 2012), mental simulation (e.g., Gerlach et al., 2011), social WM (e.g., Meyer et al., 2012), social interactions (e.g., Iacoboni et al., 2004), and task preparation (e.g., Koshino et al., 2011, 2014; Liang et al., 2016). For example, Koshino et al. (2011, 2014) investigated how the DMN and FPN regions are co-activated in a verbal WM task. It was hypothesized that both the DMN and FPN regions would show activation during the preparation period because they are both related to task preparation. However, during task execution, the FPN regions would exhibit activation, whereas the DMN regions would show deactivation. The results confirmed the hypothesis. Both the DMN and FPN regions were activated during task preparation, suggesting that the DMN and FPN might cooperate with each other. However, during task execution, the FPN regions were activated but the DMN regions were deactivated, suggesting that allocation of processing resources between the DMN and FPN could be dynamically modulated depending on resource demands of the task. Also, research showed that DMN activity during memory maintenance is associated with subsequent forgetting (e.g., Santangelo and Bordier, 2019).

It is well known that, in general, cognitive functions decline with increasing age (e.g., Hasher and Zacks, 1988; Park and Reuter-Lorenz, 2009; Grady, 2012; Cabeza et al., 2018; Salthouse, 2019). Research has shown mixed results in regard to the relationship between declines of cognitive functions and brain activation, as some reported decreased brain activation (e.g., Grady et al., 1995; Jonides et al., 2000; Logan et al., 2002), whereas others reported age-related overactivation (e.g., Grady et al., 1994; Reuter-Lorenz et al., 2000; Cabeza et al., 2004).

Several theories have been proposed to explain the age-related cognitive decline. One of them is called the neural dedifferentiation hypothesis (e.g., Anstey et al., 2003; Park et al., 2010; Hülür et al., 2015; La Fleur et al., 2018; Koen et al., 2020; Malagurski et al., 2020), which maintains that activities in older brains are more distributed across overlapping neural populations; and therefore, less distinct from each other, whereas young brains tend to form sparse representations of information. Age-related neural dedifferentiation was found in the posterior regions of the brain, such as the occipital lobe and temporal lobe, especially with stimuli including scene (e.g., Park et al., 2004; Voss et al., 2008; Zheng et al., 2018; Koen and Rugg, 2019a,b) and face (e.g., Park et al., 2004; Voss et al., 2008; Carp et al., 2010; Park et al., 2012). Dedifferentiation is also observed during associative encoding of memoranda, with less dedifferentiation (i.e., greater specificity) correlated with better memory (e.g., Grady, 2012). Neural dedifferentiation is thought to reflect an impairment of allocation of neural resources, and to compromise the accuracy of neural representations and processes (e.g., Li et al., 2001; Li and Rieckmann, 2014). Also, evidence for both compensation and dedifferentiation was found in different brain regions within the same individuals (e.g., Carp et al., 2010; Du et al., 2016), which is consistent with the claim that overactivity can provide compensation that offsets the adverse effects of neural decline in the aging brain. In other words, neural dedifferentiation might be associated with weaker within-network coherence and increased correlation among unrelated networks or decreased anti-correlation among competing networks. However, evidence for the existence of age-related cognitive dedifferentiation is mixed (e.g., La Fleur et al., 2018), as some studies have not found evidence that correlations between different measures of cognition increase with age (e.g., Payer et al., 2006; Berron et al., 2018).

The term dedifferentiation has been used in contrast to the findings that cognitive abilities differentiate (i.e., become less correlated) during child development (e.g., Li et al., 2004). Also, the effects of dedifferentiation might go beyond aging, as individual differences in neural differentiation may be a determining factor of cognitive performance throughout the lifespan. In other words, individuals with low neural differentiation may have poorer cognitive performance than similarly aged individuals with higher levels of differentiation (e.g., Song et al., 2008; Sherman et al., 2014). Research has shown that children tend to show positive correlations between FPN and DMN (e.g., DeSerisy et al., 2021), and the anticorrelation between these regions, typical of maturity, tends to develop over time. For example, Chai et al. (2014) showed that children (ages 8–12) exhibit positive connectivity, adolescents (ages 13–17) show mixed positive and negative connectivity, and adults (ages 18–24) show negative (anticorrelated) connectivity. The youth with more mature (i.e., anticorrelated) FPN-DMN connectivity demonstrated higher IQ. These results indicate dynamic network segregation (differentiation) of these networks from childhood to early adulthood.

In regard to the relationship between age-related decline in cognitive functions and brain activation, research has shown that the elderly tends to show decreased prefrontal activation during cognitive tasks (e.g., Cabeza, 2002; Reuter-Lorenz and Lustig, 2005), whereas other studies have reported greater activity and greater deactivation in the anterior brain regions. Several theories have been proposed to explain age related differences in brain activation (e.g., Festini et al., 2018), including the Hemispheric Asymmetry Reduction in Older Adults (HAROLD) model (e.g., Cabeza, 2002), the Posterior–Anterior Shift in Aging (PASA) account (e.g., Davis et al., 2008; Dennis and Cabeza, 2008), the Compensation-Related Utilization of Neural Circuits Hypothesis (CRUNCH) (e.g., Reuter-Lorenz and Lustig, 2005; Reuter-Lorenz and Cappell, 2008), the Scaffolding Theory of Aging and Cognition (STAC and STAC-r) (e.g., Park and Reuter-Lorenz, 2009; Reuter-Lorenz and Park, 2014), and the Default-Executive Coupling Hypothesis of Aging (Spreng and Turner, 2019).

According to the HAROLD model (e.g., Cabeza, 2002; Cabeza and Dennis, 2012), older adults show less lateralized prefrontal activity than younger adults while performing cognitive tasks. In other words, older adults tend to show bilateral activation of the prefrontal cortex whereas younger adults tend to show unilateral prefrontal activation. This reduction of asymmetry was thought to reflect either compensatory processes or dedifferentiation.

The PASA model (e.g., Grady et al., 1994; Davis et al., 2008; Dennis and Cabeza, 2008) is based on the observation that older adults tend to show less activation in the posterior (e.g., occipital) brain regions, along with greater activation in the anterior (e.g., frontal) brain regions compared to younger adults. The age-related frontal overactivation is typically positively correlated with performance and negatively correlated with occipital activity (e.g., Davis et al., 2008), suggesting that the prefrontal overactivity might provide compensation that offsets the adverse effects of neural decline in the aging brain, including dedifferentiation or less efficient neural circuitry. Also, Deng et al. (2021) showed that older adults exhibited greater functional integration between PFC and other brain networks, and the increase in PFC integration was associated with better task performance. They suggested that PFC reconfiguration in older adults might compensate for reductions of the MTL (Medial temporal lobe) functions, which is core region of episodic memory. It has also been shown that older adults with higher working memory capacity exhibited higher level of network integration in difficult tasks (e.g., Crowell et al., 2020).

Age-related cognitive decline is also associated with changes in activities of brain networks. Research has shown that the DMN exhibits a significant reduction of activity at rest with age and weaker deactivation during tasks (e.g., Grady et al., 2006; Damoiseaux et al., 2008; Biswal et al., 2010; Zhang and Raichle, 2010; Ferreira and Busatto, 2013; Dennis and Thompson, 2014; Mak et al., 2017). These patterns of activation may reflect a decline in switching from a default mode to a task mode (e.g., Reuter-Lorenz and Lustig, 2005; Reuter-Lorenz and Cappell, 2008). Also, the failure of suppression of DMN during tasks is related to lower performance on some cognitive tasks (e.g., Persson et al., 2007; Damoiseaux et al., 2008). Therefore, it seems possible that another cause of increased frontal activity in older adults is a failure to shift attentional resources from the DMN to task relevant networks (e.g., Reuter-Lorenz and Lustig, 2005; Reuter-Lorenz and Cappell, 2008). However, some studies have shown increased activity at rest in frontal DMN regions of elderly adults, which has been interpreted as reflecting compensation, that is, an attempt to compensate for the decrease of resting-state activity in posterior DMN areas (e.g., Davis et al., 2008).

Research on functional connectivity in older adults has shown mixed results. Some studies found that older adults showed reduced functional connectivity within the DMN compared to younger adults (e.g., Greicius et al., 2003; Biswal et al., 2010; Sambataro et al., 2010; Zhang and Raichle, 2010; Ferreira and Busatto, 2013; Dennis and Thompson, 2014; Sala-Llonch et al., 2015; Ng et al., 2016; Damoiseaux, 2017). Also, some studies have reported reduced functional connectivity in the FPN in older adults compared to younger adults (e.g., Andrews-Hanna et al., 2007; Voss et al., 2010; Marstaller et al., 2015; Ng et al., 2016). By contrast, Oschmann et al. (2020) showed significant reduced functional connectivity within the FPN and SN but detected no significant changes within the DMN. These results might suggest a progressive loss of functional specialization (dedifferentiation) with aging.

Some studies reported that the elderly tend to show increased internetwork connectivity during resting state, which suggests decline of neural segregation (Dedifferentiation) (e.g., Chai et al., 2014; Geerligs et al., 2015). Other studies also reported that older adults showed increased connectivity between the FPN and DMN. For example, Li et al. (2015) performed a meta-analysis and found that older adults had increased connectivity in the FPN and DMN, with the FPN showing a relationship with cognitive performance. Other studies with older adults have shown increased correlation and reduced anti-correlation between FPN and DMN compared to young adults (e.g., Biswal et al., 2010; Ferreira and Busatto, 2013; Chan et al., 2014; Geerligs et al., 2015). Furthermore, Ng et al. (2016) found that connectivity exhibits a u-shaped function with age. The younger elderly show a decrease in functional connectivity, whereas the older elderly show an increase in functional connectivity. They attributed this pattern to a compensation mechanism. When the elderly start showing an age-related decline of functional specialization (dedifferentiation) within the DMN and FPN, younger elderly might still be able to maintain cognitive function by recruiting additional resources (e.g., Reuter-Lorenz and Cappell, 2008; Daselaar et al., 2015). However, the adaptive mechanisms might fail for older elderly, resulting in increased DMN-PFC connectivity. Also, research showed that the elderly who exhibited more pronounced network changes between a resting and task state had better executive control performance (e.g., Gallen et al., 2016).

**Figure 1:**
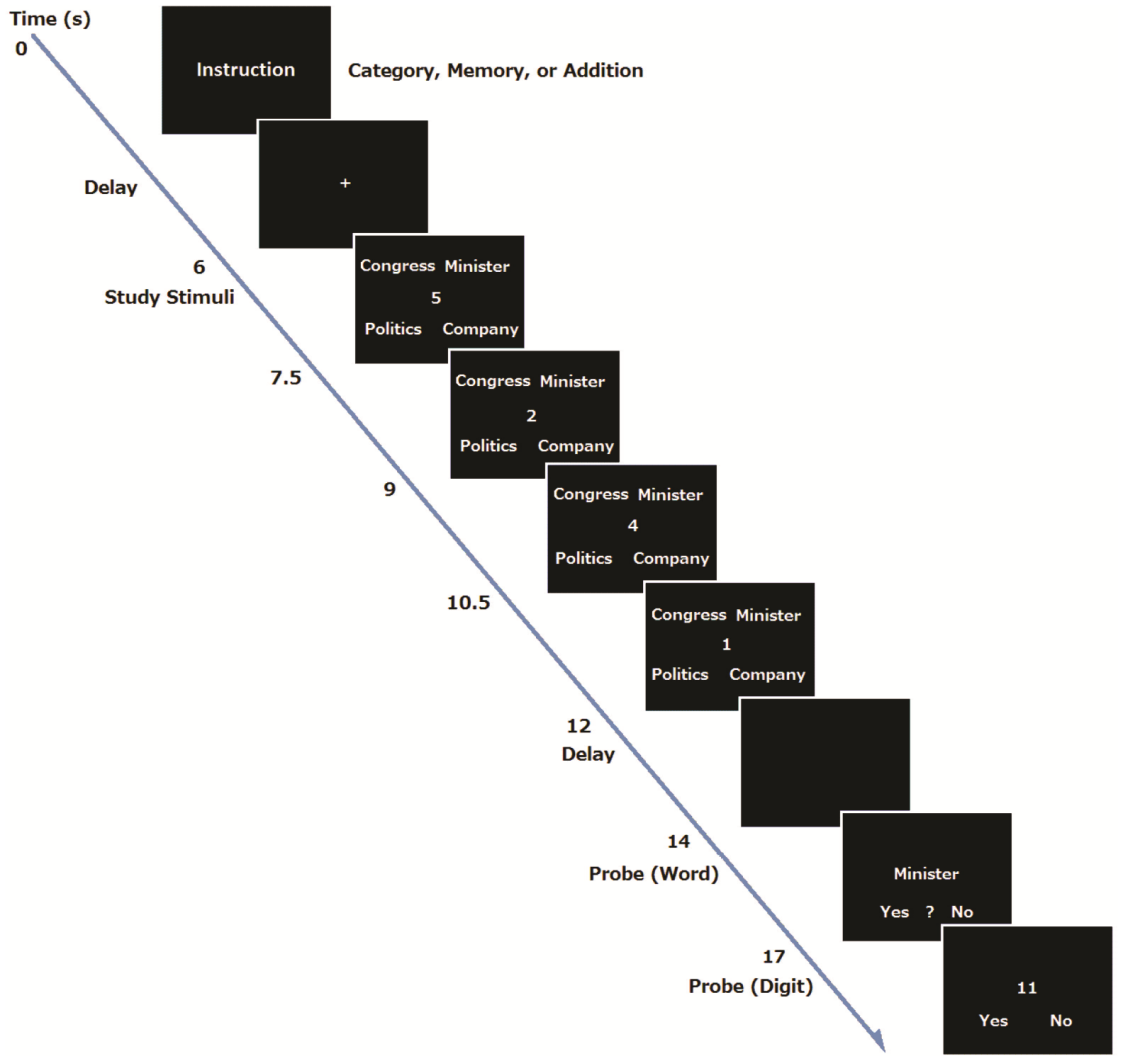
An example of trial sequence.

Here, we investigated how age-related decline in the elderly is associated with cooperation and competition between the DMN and FPN within a single task, using the working memory task from Koshino et al. (2014) (Figure 1). We hypothesized that there would be a positive correlation (cooperation) between the DMN and FPN during task preparation, and anti-correlations (competition) during task execution in both the young and elderly participants. However, we also hypothesized that the elderly participants would show weaker correlation within the DMN during preparation and execution, but stronger correlation within the FPN, especially during task execution, which would suggest compensation. In addition, we expected that the elderly participants would show lower correlation between the FPN and DMN during task preparation and lower anticorrelation between them during execution.

## Methods

### Participants

For the young group, 33 students from Kyoto and Osaka area, (7 females, mean age = 23.8, Range = 19–33) participated in the experiment. Three participants were excluded from data analysis because of excessive head motion, resulting in a total of 30 participants. For the elderly group, 30 participants (7 females, Mean age = 71.8, Range = 65–85) were recruited from Kyoto and Osaka area, through a local agency. They were screened for cognitive performance using a Japanese version of Mini-Mental State Examination (MMSE, Mean = 27.8, SD = 1.42, Max = 24, Min = 30), (Mori, 1985). Eleven participants were excluded from the analysis because of excessive head motion and missing data.

All participants had normal or corrected-to-normal vision. The participants gave written informed consent approved by the institutional review board of the Advanced Telecommunications Research Institute International (ATR) and Center for Information and Neural Networks (CiNET), and Osaka University, and were paid to participate in the study.

### Stimuli and design

We set up a Single Task condition and two types of dual task condition (Memory and Category conditions) for the young participants. Basically, these were the same tasks used in Koshino et al. (2014). In the two task conditions, four single digits were presented successively one at a time at the center of the screen and participants were required to add the digits (addition). Four words were presented at the same time in the four corners of the display. In the Memory condition, participants were asked to perform the addition task and to memorize four words. In the Category condition, participants were required to perform the addition task, and to find an odd item that did not form a group with the other three words. The stimulus words were taken from the subtest of the Kyoto-University NS Intelligence Scale (Kuraishi et al., 1955). In the Single Task condition, participants were only required to add the four digits.

For the elderly group, the dual task condition was slightly modified so that they can show essentially the same level of performance as the young group (e.g., Smith et al., 2001). Specifically, the Category condition was dropped, and the number of words was reduced from four to three.

### Procedure

A procedure was basically the same as our previous study (Koshino et al., 2014). At the beginning of each trial for the young group, the instructions for the task condition (addition, addition and memory, or addition and category) were visually presented for 3 s, and subsequently a 4 s delay was inserted (Preparation period). For the Category and Memory conditions, the participants were told to prepare for the task by forming a task set, whereas they did not receive such instruction for the Single Task condition. Then an execution period began, where the four words were presented for 6 s, and four single digits also appeared one at a time at the center of the display for 1.5 s each. The font size of words and digits was 36 points. Participants had to add the digits and remember the final answer for later recognition. For the Single Task condition, the participants were told to ignore the stimulus words, and just to perform the addition.

After the presentation of four words and digits, a fixation point was shown for 2 s, followed by a probe word that appeared at the center of the screen for 3 s. For the Single task condition, the participants were told to determine the number of characters in the probe word. The participants were told to press the left button when the probe word consisted of one character, the center button when the word consisted of two characters, and the right button when the word consisted of three characters. There was no word recognition task in the Single Task condition. For the Memory condition, the participants were told to judge whether or not the probe word was included in the four words presented previously. The participants pressed the left button when the word was presented, the center button when they were not sure whether the word was presented, and the right button when the word was not presented. For the Category condition, the participants had to judge whether the probe word was an odd item that did not form a group with the other three words. The participants pressed the left button when the word was a member of the group, the center button when the word was not part of the group, and the right button when the word was not presented. In all conditions, after the word judgment, a two-digit number appeared in the center of the screen, and the participants were required to judge whether the two-digit number was the correct answer to the addition problem within 3 s. They were told to press the left button if the two-digit number was the correct answer and the right button if the number was a wrong answer. After each trial, an inter trial interval of 6, 8, or 10 s was randomly inserted. There were 48 trials (16 trials for each condition), presented in a random order. A one-minute break was inserted every 16 trials. Stimulus presentation and behavioral data collection were controlled with the Presentation software (Neurobehavioral Systems Inc., Albany, CA, United States). Each participant received a practice session before the MRI session. There were 14 trials in total: 6 trials in the Category condition, 4 trials in the Memory and single task conditions. They were presented in a random order. An example of the trial sequence is shown in Figure 1.

For the elderly group, the dual task condition was modified so that they would show essentially the same level of performance as the young group (e.g., Smith et al., 2001). At the beginning of each trial, the instructions for the task condition (addition only, addition and memory) were visually presented for 4 s, and subsequently a six-second delay was inserted (Preparation period). Then an execution period began, where the three words were presented for 4.5 s, and three single digits also appeared one at a time at the center of the display for 1.5 s. The font size of words and digits was 48 points. Participants had to add the digits and remember the final answer. For the memory condition, they were also asked to remember three words for later recognition. After a presentation of three words and three digits, a fixation point was shown for 1.5 s. Then for the Single task condition, a probe letter appeared at the center of the screen, and the participants were told to respond to the direction of the letter (left or right) within 6 s. For the dual task condition, a probe word was presented, and the participants were asked to decide if the word was included in the three words they remembered. Then a two-digit number was presented, and the participants were required to judge whether the two-digit number was the correct answer to the addition problem.

### fMRI data acquisition and analysis

fMRI data acquisition and analysis were also basically the same as Koshino et al. (2014). Whole brain imaging data were acquired on a 3 T whole-body magnetic resonance imaging scanner (MAGNETOM Trio, A Tim System (3 T), Siemens) at ATR and CiNET. For functional imaging, a gradient-echo echo-planer imaging sequence was used with the following parameters: a repetition time (TR) = 2000 ms, an echo time (TE) = 30 ms, a flip angle = 80°, a field of view (FOV) = 192 mm × 192 mm, and pixel matrix = 64 × 64, with 3 × 3 × 5 mm voxels. 30 slice images were taken with 5 mm slice thickness in an oblique-axial plane.

After collection of functional images, T1-weighted images (191 slices with no gap) were collected for anatomical co-registration, using a conventional spin-echo pulse sequence (TR = 2,250 ms, TE = 3.06 ms, flip angle = 9°, FOV = 256 mm × 256 mm, and pixel matrix = 256 × 256, with voxel size 1 × 1 × 1 mm). After image construction, we analyzed functional images with SPM8 (Wellcome Department of Cognitive Neurology, University College London, United Kingdom). Preprocessing included slice timing correction, motion correction, normalization to EPI and spatial smoothing with an 8-mm Gaussian kernel. In a statistical model, we included separate.

covariates for the instruction of each condition (Category, Memory, and Addition), and one covariate for the presentation of word stimuli. These covariates were convolved with a hemodynamic response function (HRF).

The event duration for the preparation period was 0, whereas that for the execution period was 6. The event durations were selected based on the expected length of cognitive processes.

We interviewed the participants after the scan, and most of them reported that they selected their strategies in response to the instruction. Therefore, preparation seems to be implemented at the beginning of the preparation phase. There were three regressors for the execution period, corresponding to the experimental conditions. There was no significant correlation among regressors between the preparation period and execution period in each condition, indicating no collinearity among regressors. An FDR (*p* = 0.01) and an extent threshold (10 voxels) were used. There was no difference between the Memory and Category conditions in fMRI activation and the accuracy of performance. During the post-experimental interviews, the participants reported that they used a strategy in which they memorized all stimulus items for both the Category and Memory conditions, and tried to find an odd item at the time of response for the Category condition. This strategy seemed to cause the same pattern of performance between the Category and Memory conditions during the preparation and execution periods; and therefore, the Category and Memory conditions were combined. Then the Dual Task and the Single Task (addition only) conditions were compared in the analyses.

### Regions of interests (ROI) and percent signal change

We examined patterns of activation and deactivation across the time course in the major DMN and FPN regions compared to their own baseline (the onset of the instruction). Eight functional ROIs (four for the DMN and four for the FPN) were defined based on the fMRI activation data during the preparation and execution periods, following the same method as our previous study (Koshino et al., 2014) and with reference to the coordinates of the ROI regions in previous studies (e.g., Cole et al., 2013). A sphere was created for each cluster with a radius of 3–4 mm to maximize the coverage of the region. The functional ROIs were all bilateral, including the medial prefrontal cortex (MPFC, BA10), posterior cingulate cortex (PCC, BA31) for the DMN. For the FPN, the dorsolateral prefrontal cortex (DLPFC, BA46) and a posterior inferior region of the parietal lobe (IPLp, BA40) were selected. ROI coordinates are shown in Table 1. The activation time course for each ROI was then extracted separately for each participant for each condition using the MarsBaR (Brett et al., 2002). We computed a percent signal change (psc) for each ROI relative to the onset of the instruction (0 s) to examine activation and deactivation compared to its own baseline. Then we computed a 99% confidence interval for each data point to examine whether or not it is different from the baseline (psc = 0). In order to compute functional connectivity, we computed the time course data for each participant, and then computed the average for each ROI. Then correlation coefficients were calculated between ROIs.

**Table 1:** Coordinates of the ROI regions and their corresponding areas.

		Talairach coordinate			
		*x*	*y*	*y*	Area
1	MPFC_L	–7	46	–2	BA10
2	MPFC_R	8	49	1	BA10
3	PCC_L	–6	–53	27	BA31
4	PCC_R	8	–46	30	BA23
5	LTa_L	–51	–1	–20	BA38
6	LTa_R	44	10	–22	BA38
7	HF_L	–32	–39	–9	HF
8	HF_R	26	–38	–6	HF
9	DLPFC_L	–41	37	20	BA46
10	DLPFC_R	37	42	15	BA46
11	IPLp_L	–53	–46	41	BA40
12	IPLp_R	49	–39	42	BA40

Functional connectivity is an index of synchronization between brain regions and is measured by a correlation. In our study, we computed three types of functional connectivity. Within network connectivity for the DMN and FPN and another network connectivity between DMN and FPN. They were computed as the mean correlation among respective regions during preparation and execution separately.

## Results

### Behavioral data

**Figure 2:**
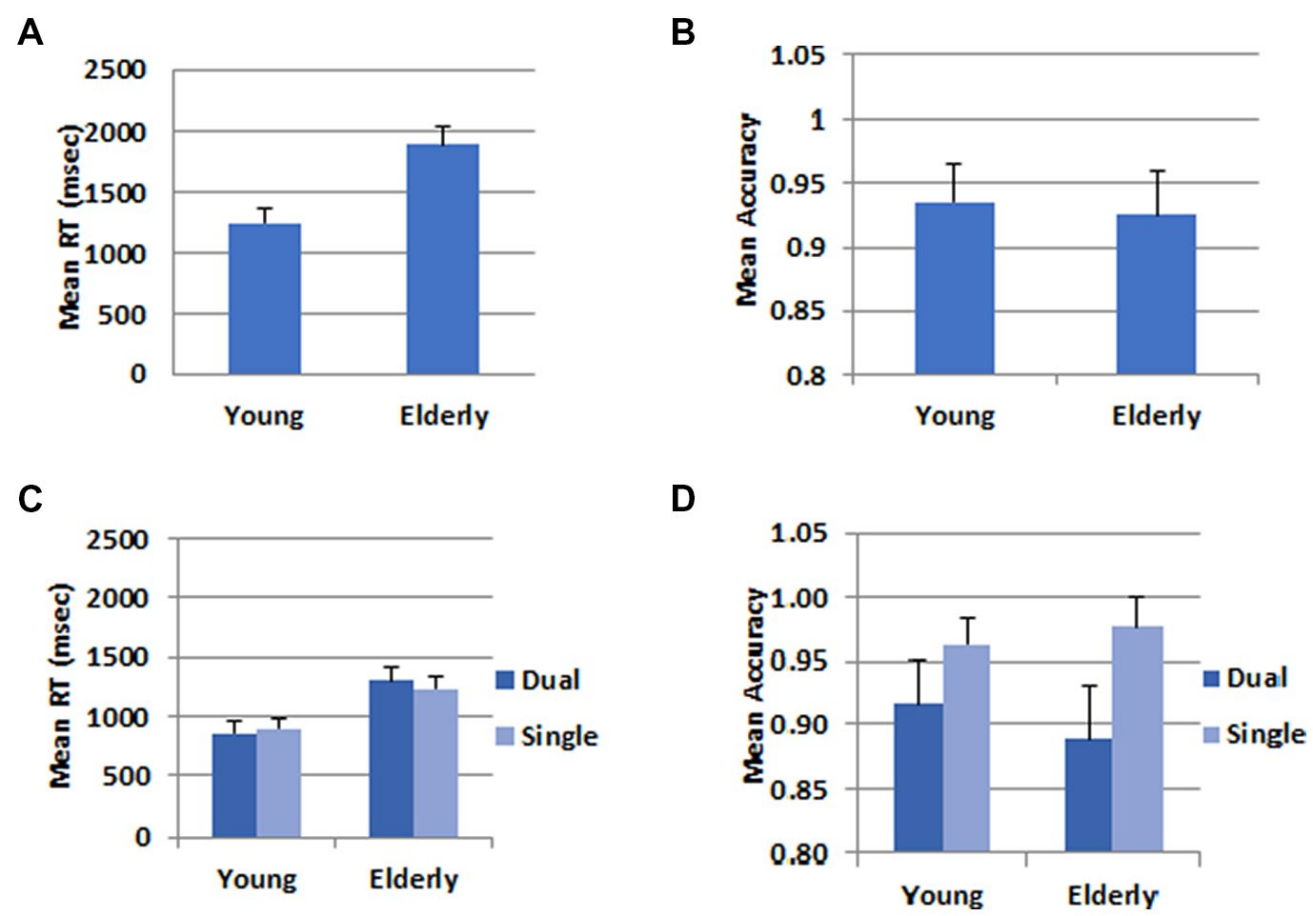
Behavioral data. **(A)** Mean RT in the WM task. **(B)** Mean accuracy in the WM task. **(C)** Mean RT in the calculation task. **(D)** Mean accuracy in the calculation task. The error bars represent the standard error.

For the behavioral data analysis, one participant was further excluded from the young group because he pressed wrong response keys. Mean Response times (RT) and accuracy rates for the WM task in the dual task condition were submitted to Analyses of Variance, and results are shown in Figure 2. The mean RT for the young participants (1247.6 ms) was significantly shorter than that for the elderly (1896.9 ms), *F*(1,46) = 44.52, *p* < 0.001, *η*_p_^2^ = 0.492; however, there was no difference between the two groups in the mean accuracy rates (Young: 0.93, Elderly: 0.92), *F*(1,46) = 0.21, *p* = 0.652.

For the addition task, the data were submitted to a 2 (Task) X 2 (Group) mixed ANOVA.

In the RT data, there was no difference between the dual (1079.8 ms) and the single task (1066.5 ms), *F*(1,46) = 0.565, *p* = 0.456. The mean RT was longer for the elderly group (1267.9 ms) than for the young group (878.5 ms), *F*(1,46) = 31.36, *p* < 0.001, *η_p_^2^* = 0.405. There was a significant interaction between task and group, *F*(1,46) = 7.71, *p* = 0.008, *η_p_^2^* = 0.144. The difference between the two groups was greater in the dual task than in the single task condition. In the accuracy data, there was a task main effect, *F*(1,46) = 22.48, *p* < 0.001, *η_p_^2^* = 0.328. The accuracy rate was higher in the single (0.97) than the dual task (0.90) condition. There was no group main effect, *F*(1,46) = 0.15, *p* = 0.701, nor a two-way interaction between task and group, *F*(1,46) = 2.22, *p* < 0.143.

### Brain activation data

**Figure 3:**
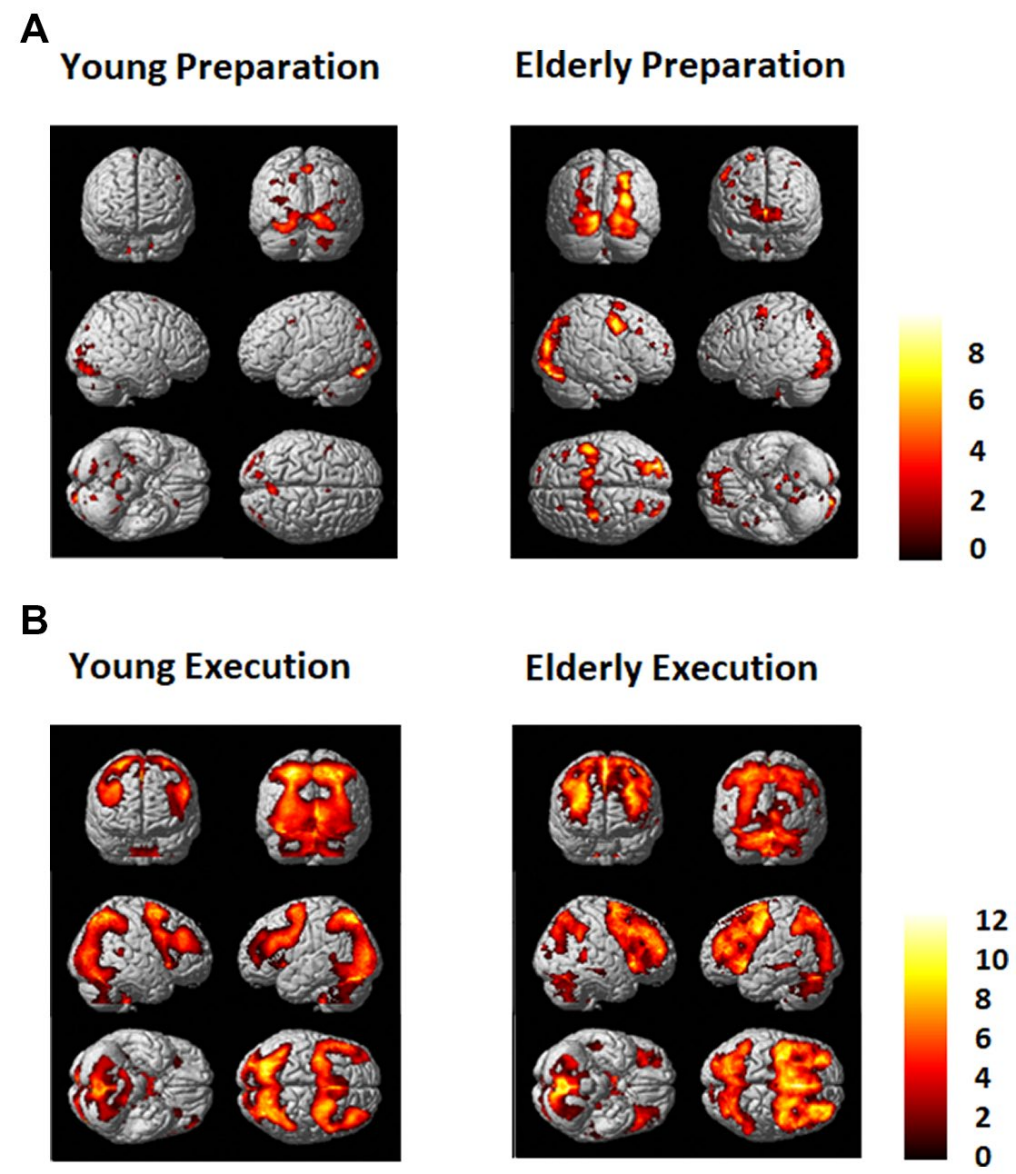
**(A)** Regions that showed greater activation in the dual task condition than in the single task condition (Dual - Single) for the young (Left) and the elderly group (Right) during **(A)** the preparation period and **(B)** during the execution period. An FDR (*p* = 0.01) and an extent threshold (10 voxels) were used.

**Figure 4:**
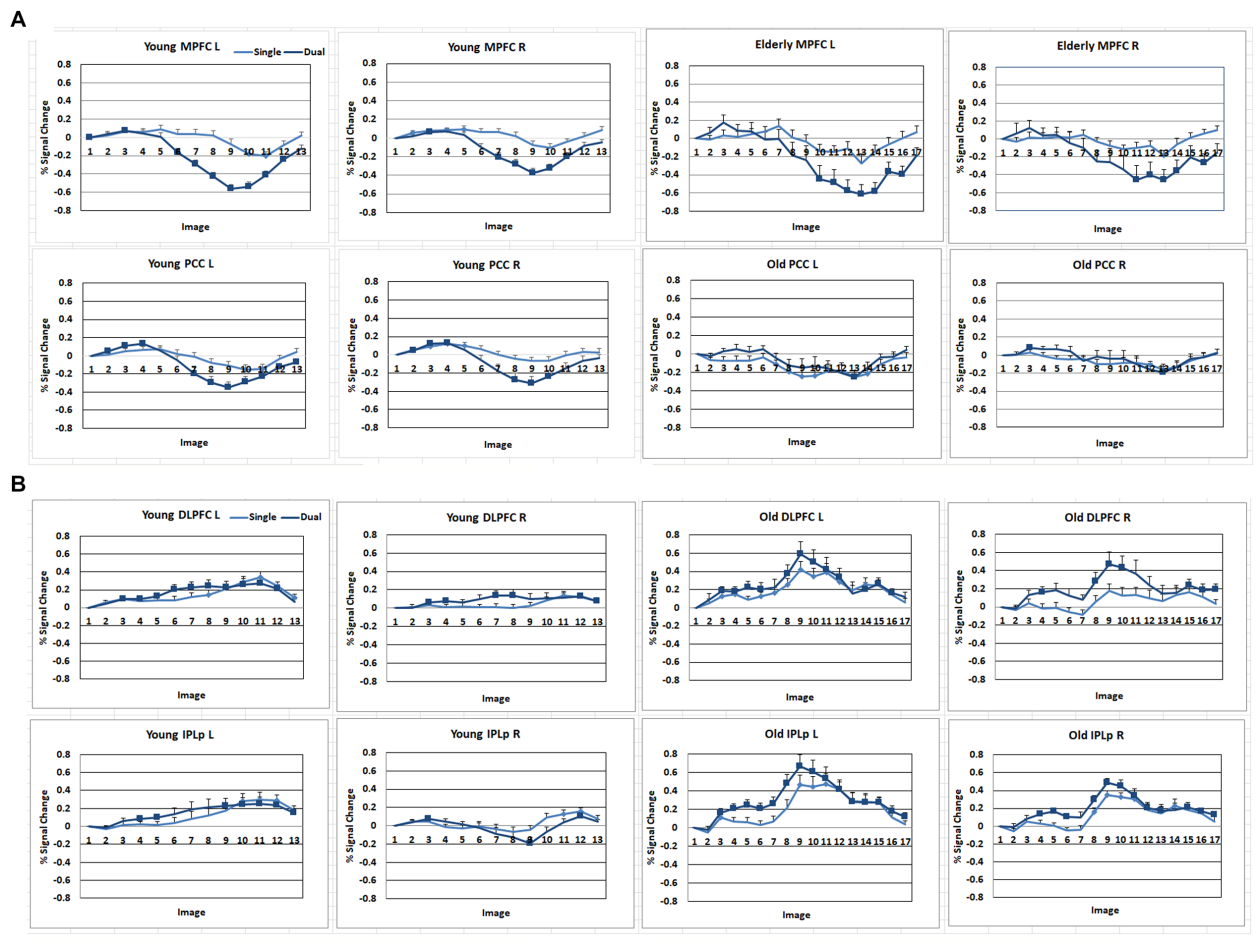
Signal change (%) of the DMN and FPN regions across the time course with the stimulus onset as a baseline. **(A)** DMN regions. MPFC, anterior medial prefrontal cortex; PCC, Posterior cingulate. **(B)** FPN regions. DLPFC, dorsolateral prefrontal cortex; IPLp, posterior inferior parietal lobe. A solid square represents the data point that the 99% confidence interval does not include zero.

Brain activation data are shown in Figure 3 and Supplementary material 1. The time course data with regions of interest (ROI) are shown in Figure 4.

As shown in Figures 3, 4, during the preparation period, the core DMN regions, including the MPFC and PCC, showed greater activation in the Dual than in the Single task condition. Within the DMN, the young group showed higher activation especially in the PCC than the elderly group, as shown in Figure 4.

During the task execution period, the DMN regions, including the MPFC and PCC, showed greater deactivation in the Dual than in the Single task condition. The elderly group showed similar deactivation to the young in the MPFC, but they did not show deactivation in the PCC, as shown in Figure 4.

The FPN regions, including the DLPFC and IPL, showed activation during the preparation period in both the elderly and young groups. However, during the execution period, the elderly showed much higher activation in the FPN than the young group.

### Differences between the young and elderly groups

**Figure 5:**
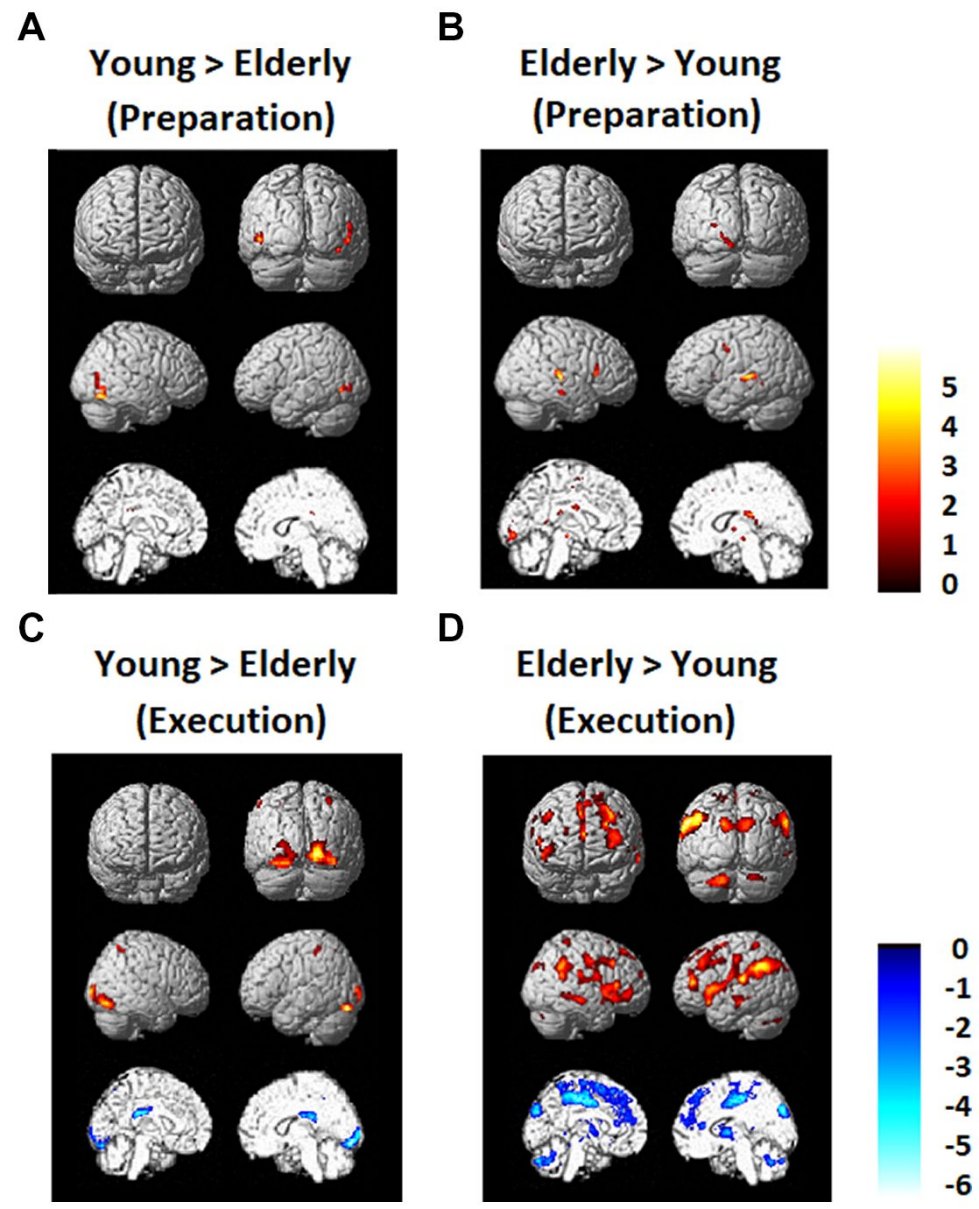
Regions that showed differences between the young and elderly groups. **(A)** Elderly > Young during preparation. **(B)** Young > Elderly during preparation. **(C)** Elderly > Young during execution. **(D)** Young > Elderly during execution.

Brain activation data exhibiting differences between the young and elderly groups are shown in Figure 5. There was not much difference between the young and elderly groups during the preparation period in the DMN and FPN in the direct comparison. However, during the execution period, the elderly group showed less activation in the occipital lobe and greater activation in the PFC, including the DLPFC and IPL. The young group also showed greater deactivation in the DMN, in the bilateral MPFC and bilateral PCC.

### Heatmap of the correlation matrix

**Figure 6:**
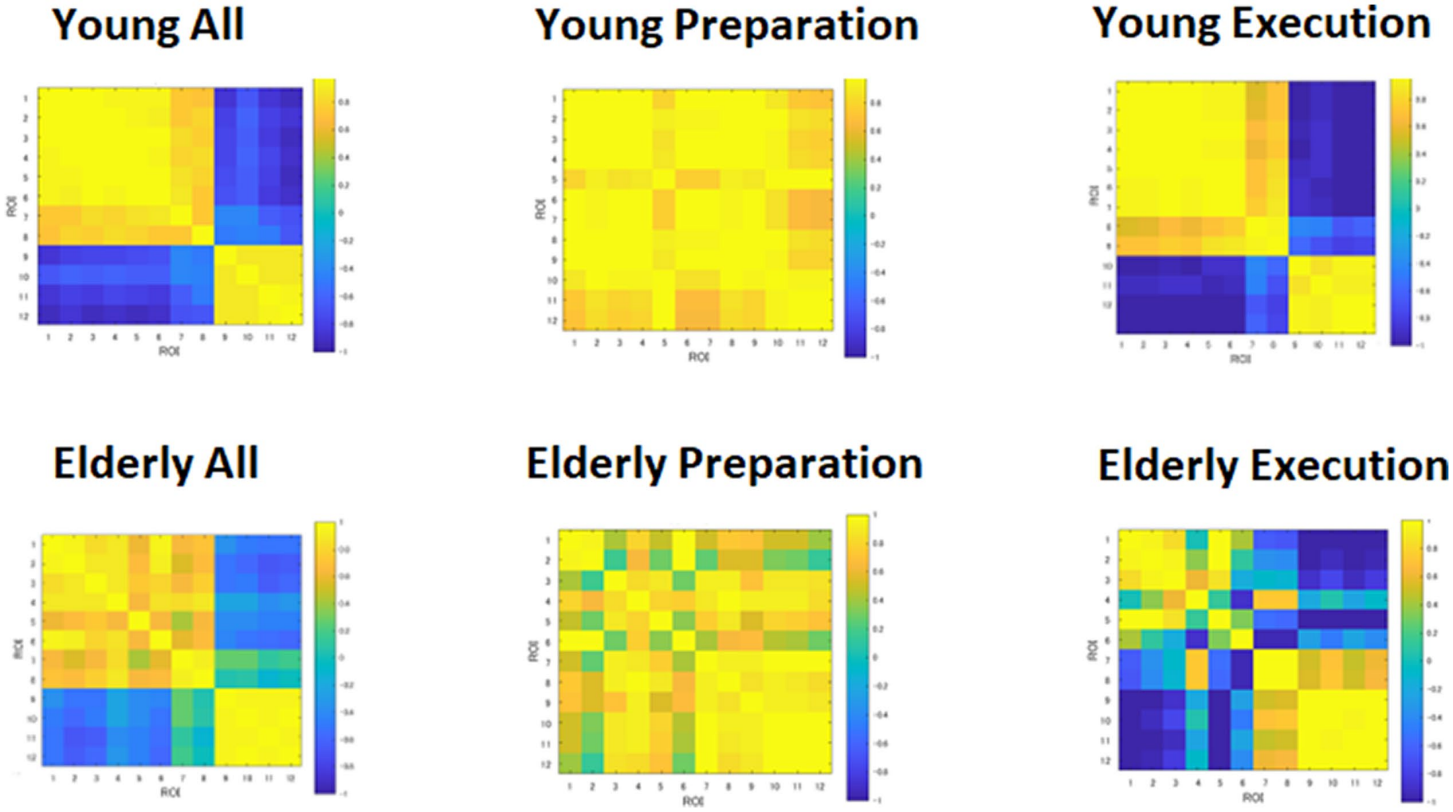
Heatmap of correlation matrices among regions of interests (ROI). DMN: 1, MPFC_L; 2, MPFC_R; 3, PCC_L; 4, PCC_R; 5, LTa_L; 6, LTa_R; 7, HF_L; 8, HF_R. FPN: 9, DLPFC_L; 10, DLPFC_R; 11, IPLp_L; 12, IPLp_R.

The heatmap of the correlation matrix (Figure 6) shows basically the same pattern as the time course data shown in Figure 4. The young group showed correlations between DMN and FPN regions during the preparation period, suggesting cooperation between the two networks. However, the young group showed anti-correlation between the DMN and FPN during the execution period, suggesting competition between the two networks. However, within-network connectivity remains high in both DMN and FPN. On the other hand, the elderly group showed lower within-network connectivity in the DMN during the preparation period, but high within-network connectivity in the DMN. The elderly group also showed lower within-network connectivity in the DMN, but they showed high within-network connectivity in the FPN during the execution period. However, the elderly group showed lower negative correlation between the DMN and FPN during the execution period.

We also computed the means of the within- and between-network correlations, for the young and elderly groups, as shown in Table 2. The young group showed greater positive correlations than the elderly group within the DMN during preparation, *t*(54) = 5.96, *p* < 0.001, and execution *t*(54) = 4.56, *p* < 0.001. In other words, network synchronization within DMN was greater for the young than the elderly group during preparation and execution. However, synchronization within FPN was not different between the young and elderly groups during preparation and execution *t*(10) = 0.46, *p* = 0.654, and execution, *t*(10) = 1.69, *p* = 0.123. However, a positive correlation between the DMN and FPN was greater for the young than elderly group during preparation, *t*(62) = 3.21, *p* = 0.002, and a negative correlation between the DMN and FPN was also greater for the young than for elderly group during execution, *t*(62) = 2.57, *p* = 0.013. In other words, between-network integration and separation were greater for the young than for the elderly group during preparation and execution.

**Table 2:** Within-and between-network correlations during preparation and execution, and group differences.

		Preparation			Execution			
		Young	Elderly	t		Young	Elderly	t
Within	DMN	0.986	0.790	5.96***		0.947	0.344	4.56***
	FPN	0.956	0.973	0.46		0.964	0.992	1.69
Between	0.948	0.808	3.21**		−0.950	−0.800	2.57*	

*p < 0.05; **p < 0.01; ***p < 0.001.

### Relation between brain activation and behavioral performance

We performed further analyses to see relationships between brain activity and behavioral performance. We computed the average brain activity during the preparation and execution periods for each ROI for the individual participants in the young and elderly groups based on the percent signal change data, and then calculated correlation coefficients with the behavioral data, including accuracy and RTs in the WM task in the dual task condition. We used the Bonferroni method for multiple comparisons. The correlation coefficients are shown in Table 3.

**Table 3:** Correlations between brain activity and behavioral performance.

	Young				Elderly			
	Prep		Exec		Prep		Exec	
	Accuracy	RT	Accuracy	RT	Accuracy	RT	Accuracy	RT
MPFC_L	0.35	−0.47	0.33	−0.04	−0.15	−0.15	0.02	0.04
MPFC_R	0.33	−0.28	0.32	0.31	−0.12	−0.28	0.05	−0.04
PCC_L	−0.15	0.09	0.15	0.26	−0.2	−0.22	−0.36	−0.14
PCC_R	−0.04	−0.22	−0.15	0.08	0.11	−0.28	0.06	−0.28
DLPFC_L	0.1	−0.06	−0.01	0.02	−0.03	−0.22	0.26	−0.12
DLPFC_R	0.1	−0.13	0.2	−0.12	0.16	−0.66*	0.27	−0.44
IPL_L	−0.11	−0.08	−0.27	0.02	0.13	−0.58*	0	−0.15
IPL_R	0.26	−0.01	−0.1	−0.1	−0.15	−0.53	−0.54	0.08

Critical *r* values with the Bonferroni method for multiple comparisons (0.05/32 = 0.00156) for each group; therefore, we used r critical values with the significance level at *p* = 0.001, and they are *r*(27) = 0.484 for the young group and *r*(17) = 0.543 for the elderly group. *Significant with the Bonferroni correction.

For the elderly group, activity in the right DLPFC during preparation was negatively correlated with RT, *r*(17) = −0.66, *p* < 0.001. The higher the activity during preparation, the shorter the RT in the WM task. Activity in the left IPL during preparation was negatively correlated with RT, *r*(17) = −0.58, *p* < 0.001. The higher the activity, the shorter the RT.

## Discussion

In the present study, we investigated how age-related decline in the elderly is associated with cooperation and competition between the DMN and FPN in the WM task. We expected to observe positive correlations (cooperation) between the DMN and FPN during task preparation, and negative correlations (competition) during task execution in both the young and elderly participants. We also hypothesized that, relative to the young participants, the elderly participants would show weaker correlation within the DMN during preparation and execution, but stronger correlation within the FPN, especially during task execution, as a form of compensation. We also expected that the elderly participants would show lower correlation between the FPN and DMN during task preparation and lower negative correlation between the FPN and DMN during execution.

The data provided partial support for the hypotheses in the present study. The results showed that the young group exhibited activation in the DMN regions, including the MPFC and PCC, during task preparation; however, these regions were deactivated during task execution, replicating our previous findings (Koshino et al., 2014). The elderly group showed weaker activation in the DMN regions during preparation. The MPFC showed some level of deactivation during execution but the PCC did not show much deactivation. Also, the direct comparison between the two group during the execution period showed basically the same pattern that the elderly group exhibited less deactivation in the DMN, especially in the bilateral MPFC and bilateral PCC. The results are consistent with the previous research that found the elderly tends to show weaker deactivation in the DMN during tasks (e.g., Grady et al., 2006; Damoiseaux et al., 2008; Biswal et al., 2010; Zhang and Raichle, 2010; Ferreira and Busatto, 2013; Dennis and Thompson, 2014), and the lack of deactivation in the DMN might be attributed to a decline in switching from a default mode to a task mode (e.g., Reuter-Lorenz and Lustig, 2005; Reuter-Lorenz and Cappell, 2008), which causes age-related cognitive decline.

Both the young and elderly groups showed activation in the FPN during task preparation and execution. However, the elderly group showed less activation in the occipital lobe but greater activation in the PFC, including the DLPFC and IPL. These results are consistent with the PASA model (e.g., Grady et al., 1994; Davis et al., 2008; Dennis and Cabeza, 2008), which claims that older adults tend to show less activation in the posterior brain regions and greater activation in the anterior brain regions compared to younger adults.

In addition to the reduced activation in the posterior regions, which is suggested by PASA, our results showed that the lack of deactivation in the PCC during execution, which might suggest a limitation of the compensatory mechanism. Brain activity in the posterior regions can be driven by both top-down (voluntary) control and stimulus-driven (automatic) control, whereas activity in the frontal regions are largely under voluntary control (e.g., Mesulam, 1981; Kim et al., 2010). Our data showed that the PCC did not exhibit deactivation during task execution, which seems consistent with the notion of a loss of automatic responses in the posterior regions. In other words, the loss of responsiveness might be observed not only in brain activation but also in brain deactivation in older adults. Therefore, our results seem to suggest that the posterior regions might lose automatic responses in both activation and deactivation with age. This failure of automatic responses in the posterior regions could be compensated by overactivation in the frontal regions, which are under voluntary control. The results of the present study are not necessarily consistent with PASA, however, because the data showed that IPL exhibited as much activation as the DLPFC during the execution period. However, the PASA model might be limited to the activity in the occipital lobe, whereas our results were based on the inferior parietal lobe. The occipital lobe is typically activated by external stimuli in a stimulus-driven fashion. By contrast, the inferior parietal lobe is viewed as a part of the phonological loop (e.g., Baddeley, 2011) in verbal working memory tasks such as the present study. Therefore, the inferior parietal lobe can be activated by the feedback loop from the prefrontal cortex in a more top-down fashion. In other words, our data may help extend the PASA model, by suggesting that some posterior regions of the brain (e.g., occipital lobe) which are activated in the stimulus-driven fashion may show less responsiveness with age, whereas other posterior regions of the brain (e.g., inferior parietal lobe) that can be activated by top-down feedback of information from the prefrontal regions might still show as much activation.

The functional connectivity analyses showed that the young group exhibited high internal correlations in the DMN during both preparation and execution, even though the magnitude of the internal correlation was the same between the young and elderly groups. The young group also showed positive correlations between the DMN and FPN regions during task preparation and high negative correlations between them during execution, as shown in Figure 6, confirming the hypothesis that DMN and FPN would cooperate during preparation but compete with each other for resources during execution. However, the elderly group showed weaker synchronization within the DMN but the same level of synchronization as the young group within the FPN during both the preparation and execution periods, as shown in Table 3. The elderly group also showed weaker negative correlations between the DMN and FPN regions during preparation and execution. These results are consistent with the dedifferentiation hypothesis (e.g., Anstey et al., 2003; Hülür et al., 2015; La Fleur et al., 2018; Koen et al., 2020; Malagurski et al., 2020). Neural dedifferentiation might be associated with weaker within-network synchronization and increased correlations among unrelated networks or decreased negative correlations among competing networks. In other words, dedifferentiation might be caused by dyssynchronization in the major networks, such as DMN. In the present study, the elderly group showed weaker within-network synchronization, and decreased negative correlations between the DMN and FPN, suggesting that age-related cognitive decline is associated with disturbance in the synchronization within the DMN, as well as the weaker negative correlation between the DMN and FPN. The elderly group, however, showed higher internal correlation within the FPN, suggesting that they put more effort to compensate for the decline of task performance.

The results of the analyses on the relationships between brain activity and behavioral performance (Table 3), showed that weak relationships between both the DMN and FPN activities and behavioral performance. For the elderly group, the higher the activity in the right DLPFC during preparation, the shorter the RT in the WM tasks. Also, the higher the activity in the left IPL during preparation, the shorter the RT in the WM tasks. The results seem to suggest that the PFN activity during preparation facilitated their responses during execution.

## Limitations

There were several limitations in the present study. One was that the attrition rate was high for the elderly group. We collected data from 30 elderly participants; however, 11 participants had to be excluded because of head motion and missing data, which limited the power of the statistical analysis.

Another limitation might be found in the screening procedure. We matched the young and elderly groups based on their behavioral performance, that there was no difference between the two groups in the accuracy rates of the WM task. However, it might be more desirable to include other matching procedures (e.g., IQ) to ensure the equivalence between the two groups.

Another limitation might be found in the fact that the present results were obtained by utilizing SPM version 8, rather than version 12, as well as employing the FDR for multiple comparisons, which is less conservative than the FWE method.

## Conclusion

In the present study, the elderly group showed lack of deactivation in the PCC, which is the posterior hub of the DMN, and a higher magnitude of activation in the IPL during execution. These results might help extend the PASA model in the following two ways. One is that age-related decline in the functions of the posterior regions might not only be seen in activation but also in deactivation, as was observed in the PCC activity during execution for the elderly participants. Another point is that some posterior regions of the brain (e.g., occipital lobe) that are activated in the stimulus-driven fashion may show less responsiveness with age, whereas other posterior regions of the brain (e.g., inferior parietal lobe) that can be activated by top-down (voluntary) information from the prefrontal regions might still show as much activation, as shown in the higher level of activation in the IPL during execution in the elderly.

In regard to functional connectivity, the present results suggest that age-related cognitive decline is associated with disturbance in the synchronization within the DMN, as well as the weaker negative correlation between the DMN and FPN. In other words, dedifferentiation might be caused by dyssynchronization in the major brain networks, such as DMN. The elderly group, however, showed higher internal correlation within the FPN, suggesting that they put more effort to compensate for the decline of task performance.

## Data availability statement

The original contributions presented in the study are included in the article/Supplementary material, further inquiries can be directed to the corresponding author.

## Ethics statement

The studies involving human participants were reviewed and approved by the Advanced Telecommunications Research Institute International (ATR) and Center for Information and Neural Networks (CiNET), and Osaka University. The patients/participants provided their written informed consent to participate in this study.

## Author contributions

HK, MO, and NO drafted the manuscript, conceived the study conceptualization, methodology, and project administration. MO and NO acquired the funding. TS, MK, and ST performed the connectivity analysis. TM and KY made the presentation program of fMRI measurements and analysis. MA and KH performed the experiments and changed the program. All authors have read and approved the final manuscript.

## Funding

This research was supported by the Japan Society for the Promotion of Science (#18H03666 ) to MO.

## Conflict of interest

The authors declare that the research was conducted in the absence of any commercial or financial relationships that could be construed as a potential conflict of interest.

## Publisher’s note

All claims expressed in this article are solely those of the authors and do not necessarily represent those of their affiliated organizations, or those of the publisher, the editors and the reviewers. Any product that may be evaluated in this article, or claim that may be made by its manufacturer, is not guaranteed or endorsed by the publisher.
