# Supplementary material for: Cooperation and competition between the default mode network and frontal parietal network in the elderly
Source: Front Psychol. 2023 May 19;14:1140399. doi: 10.3389/fpsyg.2023.1140399 (PMC10237017; doi:10.3389/fpsyg.2023.1140399)
Supplement: Supplementary file 1 [file Table_1.docx]

Supplementary materials

Table 1-a: Young preparation: Dual - Single

| set−level |  | cluster−level | |  | peak−level | |  |  |  |  |  |  |  |
| --- | --- | --- | --- | --- | --- | --- | --- | --- | --- | --- | --- | --- | --- |
| p | c | p | q | kE | p | p | q | T | Z | p | x | y | z |
| 0 | 55 | 0 | 0 | 1688 | 0 | 0.018 | 0.351 | 5.53 | 4.83 | 0 | −26 | −82 | −22 |
|  |  |  |  |  |  | 0.03 | 0.351 | 5.35 | 4.7 | 0 | 18 | −88 | −8 |
|  |  |  |  |  |  | 0.047 | 0.36 | 5.2 | 4.6 | 0 | 24 | −82 | −14 |
|  |  | 0.024 | 0.108 | 329 | 0.004 | 0.118 | 0.474 | 4.86 | 4.35 | 0 | 38 | −30 | −8 |
|  |  |  |  |  |  | 0.833 | 0.929 | 3.83 | 3.55 | 0 | 38 | −36 | −20 |
|  |  |  |  |  |  | 0.913 | 0.929 | 3.7 | 3.44 | 0 | 32 | −42 | −18 |
|  |  | 0.344 | 0.459 | 110 | 0.069 | 0.191 | 0.617 | 4.67 | 4.21 | 0 | 20 | 28 | −2 |
|  |  |  |  |  |  | 0.572 | 0.929 | 4.14 | 3.8 | 0 | 22 | 26 | 14 |
|  |  |  |  |  |  | 0.745 | 0.929 | 3.94 | 3.64 | 0 | 20 | 24 | 6 |
|  |  | 0.105 | 0.252 | 202 | 0.018 | 0.302 | 0.908 | 4.47 | 4.06 | 0 | 20 | −72 | −36 |
|  |  |  |  |  |  | 0.939 | 0.929 | 3.64 | 3.39 | 0 | 30 | −72 | −40 |
|  |  |  |  |  |  | 0.989 | 0.932 | 3.41 | 3.21 | 0.001 | 24 | −76 | −48 |
|  |  | 0.367 | 0.459 | 105 | 0.075 | 0.33 | 0.908 | 4.43 | 4.03 | 0 | −22 | 24 | 14 |
|  |  | 0.537 | 0.565 | 75 | 0.126 | 0.36 | 0.92 | 4.39 | 4 | 0 | −34 | −90 | 16 |
|  |  |  |  |  |  | 0.841 | 0.929 | 3.82 | 3.54 | 0 | −26 | −94 | 16 |
|  |  |  |  |  |  | 0.989 | 0.932 | 3.42 | 3.21 | 0.001 | −44 | −82 | 16 |
|  |  | 0.108 | 0.252 | 200 | 0.019 | 0.456 | 0.929 | 4.27 | 3.9 | 0 | 2 | −66 | 56 |
|  |  |  |  |  |  | 0.995 | 0.945 | 3.34 | 3.15 | 0.001 | 2 | −54 | 42 |
|  |  | 0.358 | 0.459 | 107 | 0.073 | 0.499 | 0.929 | 4.22 | 3.86 | 0 | 10 | −48 | 8 |
|  |  |  |  |  |  | 0.989 | 0.932 | 3.41 | 3.21 | 0.001 | 20 | −56 | 2 |
|  |  | 0.13 | 0.252 | 185 | 0.023 | 0.507 | 0.929 | 4.21 | 3.86 | 0 | −36 | −52 | −20 |
|  |  |  |  |  |  | 0.975 | 0.932 | 3.51 | 3.29 | 0.001 | −34 | −40 | −16 |
|  |  |  |  |  |  | 0.978 | 0.932 | 3.5 | 3.28 | 0.001 | −36 | −36 | 0 |
|  |  | 0.907 | 0.89 | 23 | 0.39 | 0.575 | 0.929 | 4.13 | 3.8 | 0 | −14 | 12 | 0 |
|  |  | 0.685 | 0.746 | 54 | 0.19 | 0.694 | 0.929 | 4 | 3.69 | 0 | 38 | −42 | 12 |
|  |  |  |  |  |  | 0.856 | 0.929 | 3.79 | 3.53 | 0 | 34 | −52 | 12 |
|  |  | 0.229 | 0.393 | 141 | 0.043 | 0.709 | 0.929 | 3.98 | 3.68 | 0 | 10 | −44 | −42 |
|  |  |  |  |  |  | 0.778 | 0.929 | 3.9 | 3.61 | 0 | 4 | −48 | −34 |
|  |  |  |  |  |  | 0.978 | 0.932 | 3.5 | 3.28 | 0.001 | 10 | −38 | −30 |
|  |  | 0.79 | 0.882 | 40 | 0.257 | 0.721 | 0.929 | 3.97 | 3.67 | 0 | −14 | −40 | −40 |
|  |  | 0.498 | 0.565 | 81 | 0.113 | 0.748 | 0.929 | 3.94 | 3.64 | 0 | −14 | −80 | 46 |
|  |  |  |  |  |  | 0.77 | 0.929 | 3.91 | 3.62 | 0 | −16 | −86 | 40 |
|  |  | 0.93 | 0.89 | 19 | 0.437 | 0.759 | 0.929 | 3.93 | 3.63 | 0 | 16 | −46 | −14 |
|  |  | 0.862 | 0.89 | 30 | 0.325 | 0.8 | 0.929 | 3.87 | 3.59 | 0 | −22 | 30 | −6 |
|  |  | 0.402 | 0.464 | 98 | 0.084 | 0.808 | 0.929 | 3.86 | 3.58 | 0 | −22 | −30 | −8 |
|  |  |  |  |  |  | 0.986 | 0.932 | 3.44 | 3.23 | 0.001 | −10 | −34 | −2 |
|  |  | 0.94 | 0.89 | 17 | 0.464 | 0.841 | 0.929 | 3.82 | 3.54 | 0 | 36 | −82 | 36 |
|  |  | 0.557 | 0.565 | 72 | 0.134 | 0.843 | 0.929 | 3.81 | 3.54 | 0 | 16 | −26 | −6 |
|  |  | 0.93 | 0.89 | 19 | 0.437 | 0.846 | 0.929 | 3.81 | 3.54 | 0 | −22 | −36 | 28 |
|  |  | 0.967 | 0.89 | 11 | 0.563 | 0.851 | 0.929 | 3.8 | 3.53 | 0 | 26 | −42 | −36 |
|  |  | 0.975 | 0.89 | 9 | 0.605 | 0.861 | 0.929 | 3.79 | 3.52 | 0 | 20 | −14 | 48 |
|  |  | 0.945 | 0.89 | 16 | 0.478 | 0.869 | 0.929 | 3.77 | 3.51 | 0 | −8 | −14 | 20 |
|  |  | 0.715 | 0.757 | 50 | 0.206 | 0.882 | 0.929 | 3.75 | 3.49 | 0 | −14 | −72 | −38 |
|  |  | 0.834 | 0.89 | 34 | 0.295 | 0.894 | 0.929 | 3.73 | 3.47 | 0 | −42 | −78 | 38 |
|  |  |  |  |  |  | 0.995 | 0.945 | 3.33 | 3.14 | 0.001 | −30 | −82 | 36 |
|  |  | 0.875 | 0.89 | 28 | 0.342 | 0.91 | 0.929 | 3.7 | 3.45 | 0 | −24 | −56 | 20 |
|  |  | 0.919 | 0.89 | 21 | 0.413 | 0.914 | 0.929 | 3.69 | 3.44 | 0 | 26 | −8 | 28 |
|  |  | 0.901 | 0.89 | 24 | 0.38 | 0.925 | 0.929 | 3.67 | 3.42 | 0 | 48 | −74 | 12 |
|  |  |  |  |  |  | 0.993 | 0.932 | 3.37 | 3.17 | 0.001 | 42 | −82 | 14 |
|  |  | 0.93 | 0.89 | 19 | 0.437 | 0.947 | 0.929 | 3.61 | 3.38 | 0 | −30 | −44 | −46 |
|  |  | 0.967 | 0.89 | 11 | 0.563 | 0.948 | 0.929 | 3.61 | 3.38 | 0 | 28 | −56 | −44 |
|  |  | 0.963 | 0.89 | 12 | 0.544 | 0.956 | 0.929 | 3.59 | 3.35 | 0 | 2 | 6 | 70 |
|  |  | 0.994 | 0.89 | 2 | 0.831 | 0.956 | 0.929 | 3.59 | 3.35 | 0 | 46 | 16 | −24 |
|  |  | 0.984 | 0.89 | 6 | 0.681 | 0.96 | 0.932 | 3.57 | 3.34 | 0 | −38 | −50 | 6 |
|  |  | 0.907 | 0.89 | 23 | 0.39 | 0.975 | 0.932 | 3.51 | 3.29 | 0.001 | −50 | 6 | 44 |
|  |  |  |  |  |  | 0.993 | 0.932 | 3.37 | 3.17 | 0.001 | −40 | 0 | 50 |
|  |  | 0.981 | 0.89 | 7 | 0.653 | 0.977 | 0.932 | 3.5 | 3.28 | 0.001 | 34 | −26 | 30 |
|  |  | 0.992 | 0.89 | 3 | 0.785 | 0.979 | 0.932 | 3.49 | 3.27 | 0.001 | 52 | −66 | −12 |
|  |  | 0.987 | 0.89 | 5 | 0.712 | 0.981 | 0.932 | 3.48 | 3.26 | 0.001 | 44 | −50 | −2 |
|  |  | 0.945 | 0.89 | 16 | 0.478 | 0.983 | 0.932 | 3.47 | 3.25 | 0.001 | −8 | −46 | 6 |
|  |  | 0.996 | 0.89 | 1 | 0.89 | 0.984 | 0.932 | 3.46 | 3.25 | 0.001 | 30 | −28 | 30 |
|  |  | 0.987 | 0.89 | 5 | 0.712 | 0.986 | 0.932 | 3.44 | 3.23 | 0.001 | 32 | −40 | 40 |
|  |  | 0.992 | 0.89 | 3 | 0.785 | 0.987 | 0.932 | 3.43 | 3.23 | 0.001 | 38 | −84 | 18 |

table shows 3 local maxima more than 8.0mm apart

Height threshold: T = 3.27, p = 0.001 (0.988)

Extent threshold: k = 0 voxels, p = 1.000 (0.988)

Expected voxels per cluster, <k> = 47.936

Expected number of clusters, <c> = 4.39

FWEp: 5.056, FDRp: 4.707, FWEc: 67176, FDRc: 67176

Degrees of freedom = [1.0, 47.0]

FWHM = 15.3 15.6 15.8 mm mm mm; 7.7 7.8 7.9 {voxels}

Volume: 1342592 = 167824 voxels = 326.7 resels

Voxel size: 2.0 2.0 2.0 mm mm mm; (resel = 472.89 voxels)

Table 1-b: Old preparation: Dual - Single

| set−level |  | cluster−level | |  | peak−level | |  |  |  |  |  |  |  |
| --- | --- | --- | --- | --- | --- | --- | --- | --- | --- | --- | --- | --- | --- |
| p | c | p | q | kE | p | p | Q | T | Z | p | x | y | z |
| 0 | 41 | 0 | 0 | 3094 | 0 | 0 | 0 | 8.77 | 6.71 | 0 | 16 | −88 | −8 |
|  |  |  |  |  |  | 0 | 0.002 | 6.75 | 5.62 | 0 | 18 | −82 | −18 |
|  |  |  |  |  |  | 0.009 | 0.014 | 5.78 | 5 | 0 | 24 | −94 | 8 |
|  |  | 0 | 0 | 3802 | 0 | 0 | 0.001 | 7.39 | 5.99 | 0 | −12 | −90 | −10 |
|  |  |  |  |  |  | 0 | 0.001 | 7.17 | 5.86 | 0 | −18 | −96 | −6 |
|  |  |  |  |  |  | 0 | 0.002 | 6.77 | 5.63 | 0 | −26 | −88 | 18 |
|  |  | 0 | 0 | 2519 | 0 | 0 | 0.002 | 6.75 | 5.62 | 0 | −44 | −4 | 40 |
|  |  |  |  |  |  | 0.018 | 0.026 | 5.52 | 4.82 | 0 | 30 | −4 | 46 |
|  |  |  |  |  |  | 0.123 | 0.112 | 4.84 | 4.34 | 0 | −8 | 4 | 58 |
|  |  | 0 | 0 | 1851 | 0 | 0.002 | 0.004 | 6.3 | 5.33 | 0 | 14 | 44 | −8 |
|  |  |  |  |  |  | 0.003 | 0.007 | 6.1 | 5.21 | 0 | −4 | 42 | −2 |
|  |  |  |  |  |  | 0.006 | 0.01 | 5.93 | 5.1 | 0 | 6 | 42 | −2 |
|  |  | 0.025 | 0.028 | 325 | 0.004 | 0.2 | 0.156 | 4.65 | 4.19 | 0 | 24 | −60 | 48 |
|  |  |  |  |  |  | 0.982 | 0.83 | 3.47 | 3.26 | 0.001 | 30 | −70 | 38 |
|  |  | 0.014 | 0.018 | 380 | 0.002 | 0.285 | 0.195 | 4.5 | 4.08 | 0 | 2 | −70 | 0 |
|  |  |  |  |  |  | 0.419 | 0.264 | 4.31 | 3.94 | 0 | −18 | −54 | −14 |
|  |  |  |  |  |  | 0.752 | 0.457 | 3.93 | 3.64 | 0 | −8 | −64 | −10 |
|  |  | 0.834 | 0.712 | 34 | 0.295 | 0.299 | 0.197 | 4.47 | 4.06 | 0 | −44 | 12 | −24 |
|  |  | 0.685 | 0.617 | 54 | 0.19 | 0.513 | 0.329 | 4.2 | 3.85 | 0 | 26 | 32 | 32 |
|  |  |  |  |  |  | 0.989 | 0.885 | 3.42 | 3.21 | 0.001 | 26 | 30 | 40 |
|  |  | 0.563 | 0.508 | 71 | 0.136 | 0.649 | 0.406 | 4.05 | 3.73 | 0 | −42 | 28 | 34 |
|  |  | 0.283 | 0.32 | 125 | 0.055 | 0.658 | 0.406 | 4.04 | 3.72 | 0 | −30 | −14 | −16 |
|  |  |  |  |  |  | 0.833 | 0.506 | 3.83 | 3.55 | 0 | −30 | −8 | −22 |
|  |  |  |  |  |  | 0.855 | 0.506 | 3.8 | 3.53 | 0 | −44 | −2 | −22 |
|  |  | 0.462 | 0.441 | 87 | 0.102 | 0.716 | 0.447 | 3.98 | 3.67 | 0 | −22 | −26 | −4 |
|  |  |  |  |  |  | 0.991 | 0.9 | 3.4 | 3.2 | 0.001 | −30 | −28 | 0 |
|  |  | 0.708 | 0.617 | 51 | 0.202 | 0.722 | 0.448 | 3.97 | 3.67 | 0 | 18 | −52 | −44 |
|  |  | 0.723 | 0.617 | 49 | 0.211 | 0.735 | 0.45 | 3.95 | 3.65 | 0 | 34 | −6 | −24 |
|  |  | 0.48 | 0.441 | 84 | 0.108 | 0.753 | 0.457 | 3.93 | 3.64 | 0 | −14 | −48 | −42 |
|  |  |  |  |  |  | 0.894 | 0.536 | 3.73 | 3.47 | 0 | −8 | −60 | −40 |
|  |  | 0.344 | 0.355 | 110 | 0.069 | 0.767 | 0.469 | 3.92 | 3.62 | 0 | 20 | −14 | 2 |
|  |  |  |  |  |  | 0.88 | 0.521 | 3.76 | 3.49 | 0 | 24 | −22 | 4 |
|  |  | 0.955 | 0.89 | 14 | 0.509 | 0.789 | 0.48 | 3.89 | 3.6 | 0 | −10 | 4 | 0 |
|  |  | 0.812 | 0.705 | 37 | 0.275 | 0.863 | 0.51 | 3.78 | 3.52 | 0 | 0 | −50 | −42 |
|  |  |  |  |  |  | 0.972 | 0.74 | 3.53 | 3.3 | 0 | 0 | −54 | −50 |
|  |  | 0.812 | 0.705 | 37 | 0.275 | 0.918 | 0.568 | 3.68 | 3.44 | 0 | 36 | −26 | 50 |
|  |  | 0.924 | 0.89 | 20 | 0.425 | 0.923 | 0.578 | 3.67 | 3.43 | 0 | −40 | 42 | 20 |
|  |  | 0.94 | 0.89 | 17 | 0.464 | 0.936 | 0.601 | 3.64 | 3.4 | 0 | −34 | 28 | 48 |
|  |  | 0.955 | 0.89 | 14 | 0.509 | 0.94 | 0.61 | 3.63 | 3.39 | 0 | −26 | −42 | −32 |
|  |  | 0.94 | 0.89 | 17 | 0.464 | 0.948 | 0.635 | 3.61 | 3.37 | 0 | 12 | 6 | 2 |
|  |  | 0.989 | 0.89 | 4 | 0.746 | 0.962 | 0.687 | 3.56 | 3.34 | 0 | −38 | −18 | −14 |
|  |  | 0.967 | 0.89 | 11 | 0.563 | 0.986 | 0.855 | 3.45 | 3.24 | 0.001 | 18 | −80 | 36 |
|  |  | 0.984 | 0.89 | 6 | 0.681 | 0.986 | 0.861 | 3.44 | 3.23 | 0.001 | 20 | −20 | 64 |
|  |  | 0.989 | 0.89 | 4 | 0.746 | 0.988 | 0.873 | 3.43 | 3.22 | 0.001 | 10 | −38 | −44 |
|  |  | 0.989 | 0.89 | 4 | 0.746 | 0.99 | 0.899 | 3.4 | 3.2 | 0.001 | 46 | −2 | 34 |
|  |  | 0.996 | 0.89 | 1 | 0.89 | 0.993 | 0.921 | 3.37 | 3.17 | 0.001 | 34 | −52 | −8 |
|  |  | 0.996 | 0.89 | 1 | 0.89 | 0.993 | 0.921 | 3.37 | 3.17 | 0.001 | 46 | 12 | −28 |
|  |  | 0.987 | 0.89 | 5 | 0.712 | 0.993 | 0.921 | 3.37 | 3.17 | 0.001 | 26 | −54 | −12 |
|  |  | 0.992 | 0.89 | 3 | 0.785 | 0.995 | 0.948 | 3.35 | 3.15 | 0.001 | 44 | 2 | −20 |
|  |  | 0.996 | 0.89 | 1 | 0.89 | 0.995 | 0.952 | 3.34 | 3.14 | 0.001 | 34 | −70 | 24 |
|  |  | 0.996 | 0.89 | 1 | 0.89 | 0.996 | 0.96 | 3.33 | 3.14 | 0.001 | 30 | 34 | 42 |
|  |  | 0.996 | 0.89 | 1 | 0.89 | 0.997 | 0.973 | 3.3 | 3.12 | 0.001 | −34 | −30 | 2 |
|  |  | 0.996 | 0.89 | 1 | 0.89 | 0.997 | 0.973 | 3.3 | 3.12 | 0.001 | −40 | 16 | 34 |
|  |  | 0.996 | 0.89 | 1 | 0.89 | 0.997 | 0.973 | 3.3 | 3.12 | 0.001 | −2 | −56 | −28 |
|  |  | 0.996 | 0.89 | 1 | 0.89 | 0.997 | 0.986 | 3.29 | 3.11 | 0.001 | −14 | −74 | 0 |
|  |  | 0.996 | 0.89 | 1 | 0.89 | 0.998 | 0.994 | 3.28 | 3.1 | 0.001 | −28 | −54 | −34 |
|  |  | 0.996 | 0.89 | 1 | 0.89 | 0.998 | 0.994 | 3.28 | 3.1 | 0.001 | 16 | −90 | 32 |
|  |  | 0.996 | 0.89 | 1 | 0.89 | 0.998 | 0.994 | 3.28 | 3.1 | 0.001 | −10 | −74 | 2 |
|  |  | 0.996 | 0.89 | 1 | 0.89 | 0.998 | 0.994 | 3.28 | 3.09 | 0.001 | 46 | 8 | −24 |

Table 1-c: Young Execution: Dual - Single

| set−level |  | cluster−level | |  | peak−level | |  |  |  |  |  |  |  |
| --- | --- | --- | --- | --- | --- | --- | --- | --- | --- | --- | --- | --- | --- |
| p c | c | p | q | kE | p | p | q | T | Z | p | x | y | z |
| 0.933 | 2 | 0 | 0 | 72409 | 0 | 0 | 0 | 11.95 | Inf | 0 | −26 | −66 | 52 |
|  |  |  |  |  |  | 0 | 0 | 10.25 | 7.39 | 0 | 18 | −66 | 56 |
|  |  |  |  |  |  | 0 | 0 | 10.01 | 7.29 | 0 | 30 | −66 | 44 |
|  |  | 0.982 | 0.912 | 1 | 0.912 | 0.987 | 0.984 | 3.28 | 3.1 | 0.001 | −18 | 8 | 38 |

Table 1-d: Old Execution: Dual - Single

| set−level |  | cluster−level | |  | peak−level | |  |  |  |  |  |  |  |
| --- | --- | --- | --- | --- | --- | --- | --- | --- | --- | --- | --- | --- | --- |
| p c | c | p | q | kE | p | p | q | T | Z | p | x | y | z |
| 0.078 | 8 | 0 | 0 | 67176 | 0 | 0 | 0 | 8.5 | 6.58 | 0 | −14 | −4 | 6 |
|  |  |  |  |  |  | 0 | 0 | 8.48 | 6.57 | 0 | 14 | −2 | 2 |
|  |  |  |  |  |  | 0 | 0 | 8.45 | 6.56 | 0 | −42 | 0 | 42 |
|  |  | 0.363 | 0.2 | 124 | 0.102 | 0.302 | 0.114 | 4.34 | 3.96 | 0 | 50 | −26 | −12 |
|  |  |  |  |  |  | 0.923 | 0.629 | 3.53 | 3.31 | 0 | 52 | −36 | −10 |
|  |  | 0.121 | 0.091 | 239 | 0.029 | 0.327 | 0.124 | 4.3 | 3.93 | 0 | −56 | −36 | −6 |
|  |  |  |  |  |  | 0.372 | 0.14 | 4.24 | 3.88 | 0 | −64 | −22 | −8 |
|  |  |  |  |  |  | 0.849 | 0.479 | 3.66 | 3.42 | 0 | −64 | −14 | −6 |
|  |  | 0.423 | 0.2 | 108 | 0.125 | 0.348 | 0.131 | 4.27 | 3.91 | 0 | −48 | −52 | 10 |
|  |  | 0.139 | 0.091 | 224 | 0.034 | 0.456 | 0.173 | 4.14 | 3.8 | 0 | 58 | −42 | 10 |
|  |  |  |  |  |  | 0.66 | 0.289 | 3.9 | 3.61 | 0 | 56 | −42 | 2 |
|  |  | 0.965 | 0.912 | 5 | 0.765 | 0.81 | 0.427 | 3.72 | 3.46 | 0 | −44 | 44 | −14 |
|  |  | 0.978 | 0.912 | 2 | 0.865 | 0.978 | 0.875 | 3.34 | 3.15 | 0.001 | −54 | −52 | −8 |
|  |  | 0.982 | 0.912 | 1 | 0.912 | 0.979 | 0.88 | 3.34 | 3.15 | 0.001 | 44 | 40 | −14 |
